# Discrete Missing Data Imputation Using Multilayer Perceptron and Momentum Gradient Descent †

**DOI:** 10.3390/s22155645

**Published:** 2022-07-28

**Authors:** Hu Pan, Zhiwei Ye, Qiyi He, Chunyan Yan, Jianyu Yuan, Xudong Lai, Jun Su, Ruihan Li

**Affiliations:** 1 School of Computer Science, Hubei University of Technology, Wuhan 430068, China; 102010986@hbut.edu.cn (H.P.); qiyi.he@hbut.edu.cn (Q.H.); cyyan@hbut.edu.cn (C.Y.); a2htray@outlook.com (J.Y.); sujuncs@hbut.edu.cn (J.S.); 102111083@hbut.edu.cn (R.L.); 2 Fujian Provincial Key Laboratory of Data Intensive Computing, Quanzhou 362000, China; 3 Key Laboratory of Intelligent Computing and Information Processing, Fujian Province, Quanzhou 362000, China; 4 School of Remote Sensing and Information Engineering, Wuhan University, Wuhan 430072, China; laixudong@whu.edu.cn

**Keywords:** data preprocessing, discrete missing data, data imputation, multilayer perceptron, momentum gradient descent algorithm

## Abstract

Data are a strategic resource for industrial production, and an efficient data-mining process will increase productivity. However, there exist many missing values in data collected in real life due to various problems. Because the missing data may reduce productivity, missing value imputation is an important research topic in data mining. At present, most studies mainly focus on imputation methods for continuous missing data, while a few concentrate on discrete missing data. In this paper, a discrete missing value imputation method based on a multilayer perceptron (MLP) is proposed, which employs a momentum gradient descent algorithm, and some prefilling strategies are utilized to improve the convergence speed of the MLP. To verify the effectiveness of the method, experiments are conducted to compare the classification accuracy with eight common imputation methods, such as the mode, random, hot-deck, KNN, autoencoder, and MLP, under different missing mechanisms and missing proportions. Experimental results verify that the improved MLP model (IMLP) can effectively impute discrete missing values in most situations under three missing patterns.

## 1. Introduction

With the advent of the data age, scientific and engineering practices generate explosively growing, widely available, and large volumes of data. These data contain many latent laws of development of various industries that are attracting more and more attention from academia and industry [1]. Over the past few decades, many enterprises have taken advantage of data to assist in making production decisions, achieving great benefits [2]. With the advancement of global informatization, analyzing and utilizing data play a significant role in promoting social development. However, there are some problems in the data-collection process, such as functional limitations, failures of equipment, incorrect data, temporary modification, and lack of responses to surveys [3], which generate considerable amounts of missing values in the final datasets obtained. These incomplete, unprocessed datasets may affect data analysis results, reduce data utilization, and even lead to wrong decisions [4]. Therefore, manipulating missing values in datasets is critical to improving the benefits of mining and using data, which is of great research significance.

Because of the importance of dealing with missing values in the object dataset, scholars have investigated many solutions. Generally, deleting the tuples with missing values is one of the most straightforward and commonest methods [5]. However, its performance is extremely unsatisfactory when the percentage of missing values per attribute varies considerably. Moreover, removing the tuples may make the remaining attribute values in the entire dataset less useful, as the deleted tuples may be crucial for the task at hand. Studies have shown that after removing missing data, good performance can be maintained only if the missing proportion is less than 10% or 15% [6]. To solve the mentioned problems with deleting missing values, another solution for this issue is to impute the missing values using statistical or machine learning techniques. Statistics study the collection, analysis, interpretation or explanation, and presentation of data, and statistical techniques model missing data values to fulfill data imputation. Initially, statistical techniques were utilized to find out the latent laws within the complete data to impute missing values, including the mean (mode), random, hot-deck [7], etc. Taking the mode as an example, a measure of central tendency for the attribute is applied to fill in the missing values. Due to the ease of implementation of statistical techniques, they have been successfully used in various fields and remain one of the major solutions for missing values imputation [8]. However, statistical imputation methods may downgrade in performance when the observed values are not close to the actual estimate of the missing value [9]. Machine learning techniques enable computer programs to automatically learn to recognize complex patterns and make intelligent decisions based on data. In this regard, imputation methods based on machine learning techniques develop models using different observed attributes to effectively fill in the missing values of the unobserved attributes [10]. Compared with the statistics-based imputation methods, machine learning techniques make missing value imputation more precise and accurate by selecting the most similar patterns or features to the actual estimates of the missing values. Consequently, machine learning techniques play the same role as statistical techniques in reconstructing a complete dataset [11].

Machine learning techniques have formed the bases of a series of efficient frameworks for data processing over the past few decades, such as the k-nearest neighbors (KNN) [12,13], artificial neural network (ANN) [14], support vector machine (SVM) [15], autoencoder (AE) [16], and multilayer perceptron (MLP) methods [17], some of which have also been applied in missing value imputation. For instance, Sanjar et al. developed a prediction model for house prices with correlations between features utilizing the KNN algorithm. It outperformed the mentioned baseline in [13], but calculating the similarity based on KNN cost computational overhead, and the effect of the missing ratio on imputation performance was ignored. To explore the effect of the missing rate on imputation performance, Lin et al. applied deep belief networks with a feature extraction strategy for missing values imputation, conducting experiments with different missing proportions ranging from 1% to 15% [18]. Wang et al. developed a transfer learning model with an additive least squares SVM to improve the classification performance for incomplete datasets, with which experiments were conducted using different missing proportions from 10% to 60% [15]. It has been testified that such approaches have stable performance with various missing proportions, even if the missing ratio is over 50%. Moreover, AE can learn to represent the incomplete data and infer alternative data for the missing value [16]. Pereira et al. summarized and discussed the successful cases of using autoencoders in missing data imputation during the last decade [19], which showed that denoising autoencoders provided a suitable choice for cases of missing values. Yoon et al. introduced the GAN framework to deal with missing values, and their proposed method significantly exceeded some state-of-the-art imputation methods [20]. Nonetheless, all the above research principally focused on continuous missing data, and the final imputation performance is affected by the data size. MLP is another representative deep learning framework for missing data imputation [21], and it has shown its superiority in cases of large-scale of data or unstructured data. For instance, Gad et al. and Cheng et al. took advantage of MLP as a framework for handling climate and medical data [22,23], respectively. Because of its simplicity and efficiency, MLP has been investigated further to improve the performance of missing value imputation in recent years. Jung et al. introduced a novel missing value imputation scheme utilizing a bagging ensemble of multilayer perceptrons, which provided superior performance in the case of electricity consumption data [24]. Śmieja et al. proposed a variation of origin MLP for several incomplete datasets, with missing ratios from 0.25% to 23.8%, which verified that the MLP imputation method was widely applicable in distinctive domains [25]. In [26], experiments testified that the MLP method was optimal for numerical and mixed datasets in terms of classification accuracy, while the classification and regression tree (CART) method performed well for categorical datasets.

Though imputing the missing data based on an MLP has desirable performance in specific applications, there are still several challenges. First, considerable training time and computational cost are required to maintain the imputation performance, which might reduce its practicality to some extent. Second, real-world datasets are composed of continuous and discrete data in most cases, namely, there are both continuous and discrete missing values. However, many studies only paid attention to continuous data, while little research concerned discrete missing data [27,28]. Although some mentioned methods could theoretically handle discrete missing values with some definite manipulations, they were always slow or inefficient for object problems. Finally, there are actually three missing mechanisms according to the occurrence of missing values, but most of the previous research has studied only one missing mechanism (more details will be introduced in the next section). Therefore, it is essential to improve the standard MLP method to overcome the existing problems and propose a novel imputation scheme based on an improved MLP technique for discrete missing data. The main contributions of this paper are as follows:

- An imputation scheme for discrete missing data based on a multilayer perceptron with the gradient descent algorithm and prefilling strategy is proposed.
- A performance evaluation is conducted on seven real-world datasets for three missing mechanisms compared with eight classical methods, which was mainly done for one missing mechanism in previous work. Several levels of noise are artificially added to simulate missing proportions according to the definition of missing mechanisms, and the effect of missing proportions is explored.

The paper focuses on developing an efficient imputation method for filling in incomplete datasets, especially datasets with discrete missing values. As a result, an imputation method based on an improved multilayer perceptron with the momentum gradient technique [29] is proposed to overcome the insufficiency of the basic MLP method [30], which combines different prefilling strategies for specific missing patterns. To verify the effectiveness of this method, comparisons are conducted on various statistical imputation methods and machine learning imputation methods under different missing patterns and missing proportions.

The rest of the paper is structured as follows. Section 2 introduces the fundamental conceptions of missing data, representative imputation methods, and the MLP framework. The improved MLP imputation scheme for discrete missing data is concretely illustrated in Section 3. Section 4 describes the experimental settings and presents the results of the experiments, analyzing the latent laws within the results. Lastly, Section 5 summarizes the conclusions and provides avenues for future work regarding discrete missing data imputation theory.

## 2. Materials and Methodology

Data preprocessing plays a significant role in data mining and analysis, typically including normalizing data, removing noise, dealing with missing values, etc. Because missing data generally downgrade the efficiency of data mining and analysis, much recent research concentrates on handling them to improve the quality of data based on statistical techniques, machine learning techniques, ensemble methods, etc. For instance, Emmanuel et al. discussed and summarized the classical techniques for missing data imputation [31], proposing and evaluating two methods using a missing rate of 5% to 20%. The results certified that KNN can successfully deal with missing data. However, their major experimental object was continuous data, providing insufficient comparisons with more missing rates and compared imputation methods. Unsupervised machine learning techniques are another typical way to handle missing data. Raja et al. conducted experiments on the Dermatology; Pima; Wisconsin, United States; and Yeast datasets, utilizing an improved fuzzy C-means algorithm to enhance the utilization of information [32]. Li et al. combined fuzzy C-Means and a vaguely quantified rough set to detect reasonable clustering for missing data [3]. Nevertheless, its computational cost is still a challenge for application in practice. Machine learning techniques with neural networks have increasingly shown their superior ability to handle sizeable data. To reduce the influence of missing values on prediction performance, Lim et al. developed an LSTM-based time series model for predicting future values of liquid cargo traffic with other evaluation indexes, finding that the proposed model improved the prediction performance [33]. Zhang et al. adopted an end-to-end GAN framework to handle multivariate time series data, which was combined with an encoder network to improve the prediction performance [34]. Li et al. aimed to explore random missing and continuous missing situations for dam SHM systems, proposing a combination of deep learning and transfer learning to improve the generalization of the missing data imputation scheme [35]. Many successful applications of the deep learning technique have shown that it is well suited to missing data. However, considerable training data and computational resources are needed for deep learning techniques, which may be an obstacle to their application in practice. Furthermore, their ignorance of concerning discrete missing data also downgrades their practicability.

MLP has good applicability to data processing, and it has been successfully used in missing data imputation [36,37]. Recently, the influences of missing mechanisms, missing rates, and specific applied domains have been further studied. Missing mechanisms can affect the imputation performance of different methods. To explore imputation performance in MAR, Fallah successfully utilized the MLP method to impute time series landfill gas data [38]. Śmieja et al. performed experiments with missing rates from 0.25% to 23.8% based on the MLP method for continuous data [25]. Luo et al. evaluated MLP with other competitive machine learning or statistical models for clinical data [39]. Lin et al. conducted research on the effect of data discretization for continuous data, where MLP and DBN were significantly superior to the mentioned baseline imputation methods [40]. To repair missing data for credit risk prediction, Yang et al. developed an ensemble MLP model with superior accuracy to the traditional machine learning model, which testified that repairing missing data can improve the model’s prediction ability [41]. However, more comprehensive consideration of missing mechanisms and missing rates is first required for wide application of the technique to imputing missing data. Discrete data are of great significance for studying, as insufficient processing may reduce data utilization and decision making. All of these concerns motivate us to propose a new scheme for discrete missing data to improve imputation performance, which theoretically is conducted on three missing mechanisms and five levels of missing proportions.

This section presents some fundamental concepts of missing data, especially regarding discrete missing data and missing patterns. The basic methodology of the missing data imputation technique is provided next. Finally, MLP and the gradient descent algorithm are accordingly introduced.

### 2.1. Discrete Data

**Figure 1:**
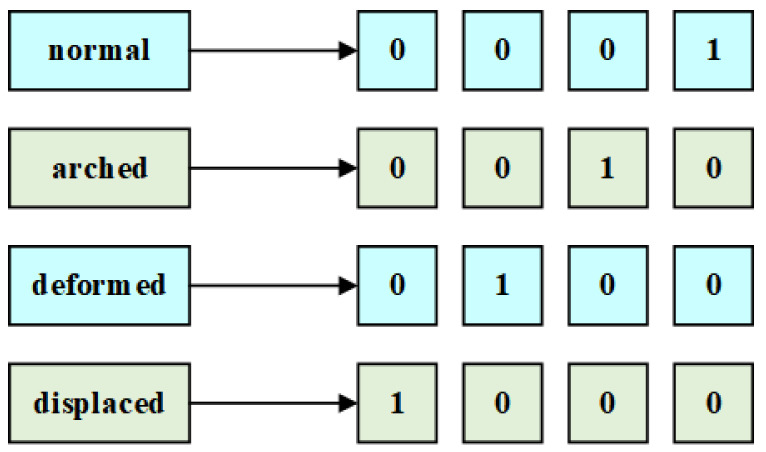
An example of the one-hot encoding technique in the Lymphography dataset.

There are two types of data, i.e., continuous and discrete data, where discrete attributes refer to attributes with a finite or infinite number of values represented with or without integers. Generally, discrete attributes include ordinal attributes, binary attributes, nominal attributes, etc. Most research concentrated on imputation methods for continuous missing data, such as regressions [42,43], decision trees [44], and deep learning techniques [42], but few studies offered solutions to handle discrete missing data. For instance, a dataset named Lymphography from the UCI Machine Learning Repository [45] used in this study contains an attribute named lymphatics, including four attribute values: normal, arched, deformed, and displaced. However, most machine learning techniques cannot directly deal with discrete missing data. If such discrete data enter an imputation model without processing, most such models will not perform well. Thus, it is essential to preprocess datasets containing discrete data. One-hot encoding is a common discretization technique based on binary coding which can effectively deal with the different types of data, expanding the feature space to some extent. Figure 1 shows the corresponding forms with one-hot encoding of the above example, where 1 represents the position of the discrete value in coding space In particular, the one-hot technique is also suitable for discrete integer values.

### 2.2. Basic Methodology for Missing Data Imputation

The missing mechanism or pattern is an inherent feature of the missing data. In [10], the missing pattern was theoretically classified into three categories, including missing completely at random (MCAR), missing at random (MAR), and not missing at random (NMAR). Specifically, MCAR means that the missing values occur randomly and do not generate deflection in the results, which have no relationship with the observed and unobserved data. MAR refers to a pattern in which missing values are related to the observed data, which suggests the missing data can be inferred from the existing data. The case in which missing data are related to the unobserved data can be classified as NMAR. This theory means that the missing values can be effectively imputed with some specific laws.

According to the definition of missing mechanisms, two primary type methods have been summarized in theory: statistics-based and machine-learning-based imputation methods. The former utilizes statistical principles to infer the missing values, and the mean and random are the two most representative approaches. Specifically, the mean imputation method chooses the mode or mean of the observed data to fill the missing values, and it has been widely applied in imputing missing values [10]. For numerical missing data, this method calculates the mean values of the observed corresponding attributes as the filling values of the missing values; for the nonnumerical case, it uses the mode as a substitute for the missing values. The random imputation method is another common solution to missing data in surveys [46]. Its imputation scheme depends on the probability of each feature value in the whole observed dataset. It randomly selects an observed value as the imputation result, which means that a more frequent value is more likely to be chosen to replace a missing value. Because machine learning techniques are highly efficient for sizeable data analysis, the second type of imputation method takes full advantage of machine learning. Several typical machine-learning-based imputation methods of filling missing values are as follows.

The k-nearest neighbors (KNN) technique can mine the latent patterns based on the similarity between samples. Therefore, the imputation method based on the KNN technique selects k complete samples closest to the missing samples from the whole complete sample set as candidate samples and takes the weighted average value of the observed values in the candidate samples as the filling value [47]. There are many metrics to determine the neighbors of the objective sample, and the Hamming distance has been chosen as the distance metric in many studies [48]. It counts the sum of the number of different positions of two strings of equal length, and its definition can be expressed as Equation (1):

(1) $$HD=\sum_{i=1}^{m}A_{i}⊗B_{i}$$

where A and B are two samples involved in the calculation. $A_{i}$ and $B_{i}$ represent the ith feature of A and B, respectively, and their values might be 1 or 0. m represents the dimension of feature space. The Hamming distance describes the similarity of different samples, and the smaller the distance, the more reliable the filling value that is obtained. Unlike the imputation method using the KNN technique, another type of imputation method based on machine learning techniques regards the filling process as a classification task, aiming to figure out a similar pattern in missing data. Generally, all the incomplete features are divided into several subgroups, where each subgroup represents a classification target. The features without missing values are fed into a specific learning model for each target. The random forest [49] and decision tree algorithms [44] are two representative methods in this category.

Deep learning is the main branch of machine learning which is especially suitable for unstructured data, such as images and text documents [50]. However, only a few missing value imputation methods for tabular or structured data have been studied. The autoencoder is a typical deep learning technique with the same number of neurons in the input and output layer [19]. Because of its special neural network structure, an autoencoder is easy to implement, as it only needs to train the weight of a single network. The implementation steps of missing value imputation with an autoencoder are presented in detail below:

Step 1: Determine the network structure according to the object dataset;

Step 2: Split the dataset into the complete subset ($D_{com}$) and the incomplete subset ($D_{miss}$);

Step 3: Take $D_{com}$ as the training set, and calculate the weights of the network;

Step 4: Prefill the incomplete subset. Take the samples in $D_{com}$ as the input of the training model, and the missing values can be filled with the predictions of the trained model.

### 2.3. Multilayer Perceptron

**Figure 2:**
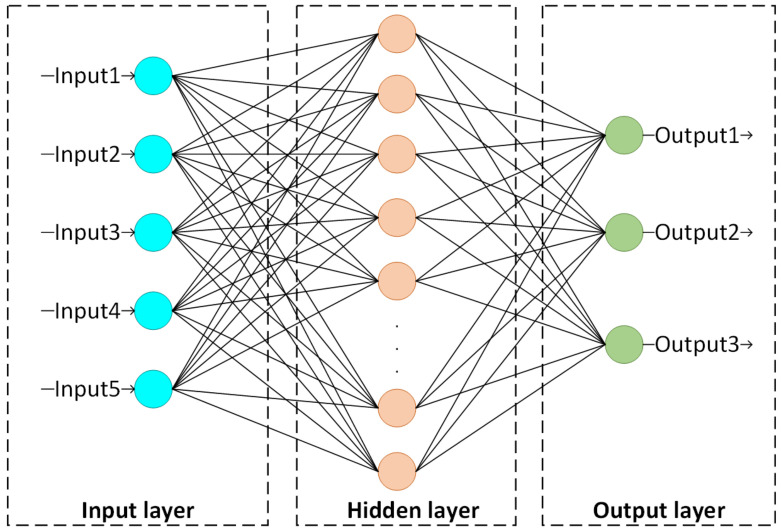
A basic MLP model framework.

The multilayer perceptron (MLP) has been widely used in sizeable data processing, especially for images and text documents [40]. In general, it is composed of an input layer, an output layer, and multiple hidden layers, and each neuron between adjacent network layers is fully connected. A standard MLP model is shown in Figure 2.

An MLP utilizes a supervised learning technique called backpropagation during the training phase [40]. As shown in Figure 2, external data are directly transmitted to the next layer without any computational processing via the input unit. The neurons in the hidden and output layers are the computational units of the network. During the training stage, the input data are first weighted and summed with the bias parameter, and then the summed value is transferred to the activation function, and the output is obtained. Finally, the output neurons output the predictions of the model. As for the weight updating and error function, the gradient descent algorithm and the cross-entropy error are commonly utilized, which are also related to the characteristics of the object dataset. Specifically, the training process is as follows.

First, the input from a specific neuron can be expressed as a multiple-dimensional vector $x_{i}$, and then the hidden unit outputs $net_{ik}$ are computed as Equation (2):

(2) $$net_{ik}=\varphi \left(\sum_{l=1}^{S}\omega_{lk}^{\left(1\right)}\cdot x_{il}+b_{k}^{\left(1\right)}\right)$$

where the superscript (1) refers to the corresponding parameters in the first layer of the MLP. $x_{il}$ indicates the lth attribute of $x_{i}$ in the whole set of S attributes. $\omega_{lk}$ is the connected weight between the lth unit in the input layer and the kth unit in the hidden layer. $b_{k}^{\left(1\right)}$ denotes the bias parameter of the kth unit in the hidden layer. The outputs summed have to activate via φ, which is a particular function, such as $\tanh\left(x\right)$, $sigmoid\left(x\right)$, or $relu\left(x\right)$. Following that, all the $net_{ik}$ values are linearly combined and transformed by an output activate function ϑ. The output unit $y_{ij}$ is computed via Equation (3):

(3) $$y_{ij}=\vartheta \left(\sum_{k=1}^{K}net_{ik}^{\left(1\right)}\cdot \omega_{kj}^{\left(2\right)}+b_{j}^{\left(2\right)}\right)$$

where the superscript (2) refers to the corresponding parameters in the second layer of the MLP, and $net_{ik}^{\left(1\right)}$ is the same as $x_{il}$ in Equation (2).

### 2.4. The Gradient Descent Algorithm with Momentum

An MLP is a typical artificial neural network, consisting of several components, including neurons, weights, biases, and activation functions. As for training an MLP model, the main task is to determine the parameters between adjacent layers, including the connected weights and biases. Regarding calculating parameters as an optimization problem, one of the solutions is to make use of gradient descent. Specifically, all parameters are stochastically initialized at first. The model is then trained iteratively, continuously calculating the gradient and updating parameters until a specific condition is met (e.g., the error is less than a threshold, or the number of iterations reaches a threshold). Though the gradient descent technique has been widely used in parameter optimization, there are still several challenges involving it to be solved. One of the challenges is to overcome the risk of falling into a local optimum. Researchers have proposed several optimization algorithms to deal with this problem, including batch gradient descent, stochastic gradient descent, and mini-batch gradient descent. On the other hand, each update manipulation of the traditional descent algorithm in the MLP training process is based on the current position, slowing the convergence speed. Therefore, momentum gradient descent (MGD), as a kind of stochastic gradient descent algorithm, is introduced into the MLP model [29]. Incorporating the MGD algorithm, the parameters can be updated via Equations (4)–(6):

(4) $$v_{\nabla \omega }^{k+1}=\beta \cdot v_{\nabla \omega }^{k}+\left(1-\beta \right)\cdot \nabla \omega^{k}$$

(5) $$v_{\nabla b}^{k+1}=\beta \cdot v_{\nabla b}^{k}+\left(1-\beta \right)\cdot \nabla b^{k}$$

(6) $$\omega^{k+1}=\omega^{k}-\alpha \cdot v_{\nabla \omega }^{k+1},b^{k+1}=b^{k}-\alpha \cdot v_{\nabla b}^{k+1}$$

where α is the learning rate; β is the momentum coefficient, and its default value is always set to 0.9. v is the momentum used to control the convergence speed, and its introduction combines all the previous ∇ω and ∇b via Equations (4) and (5).

## 3. The Proposed Method

Completing missing values using a machine learning technique aims to estimate the missing value by finding the correlations among attributes. Its goal is to develop prediction models for the missing values, which is generally regarded as a classification task. In this regard, building an accurate nonlinear prediction model for the unobserved set is the key to ensuring high imputation accuracy. The standard MLP architecture is chosen to impute discrete missing data in this study. The research framework and proposed scheme for discrete missing values are discussed in this section.

### 3.1. An Overview of the Proposed Method

**Figure 3:**
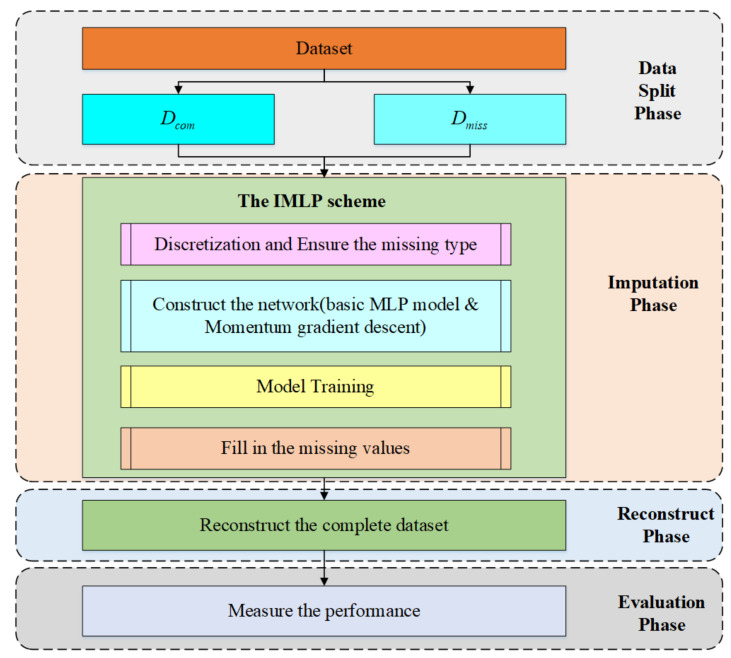
The overall workflow of the proposed IMLP imputation technique.

Before the detailed discussion of the proposed methodology, an overall framework will be presented. Figure 3 exhibits a comprehensive overview of the proposed method, in which its process can be summarized as follows:

Step 1: The object dataset is divided into two parts: the observed or complete samples as subset $D_{com}$, and the incomplete samples with missing attribute values as subset $D_{miss}$. In the study, missing values are artificially simulated using complete datasets with different ratios for the three missing mechanisms;

Step 2: Discretizing the object data and ensuring the missing type are two preliminaries of our scheme. An MLP with momentum gradient descent, called an IMLP, is applied to fill in the missing values. The IMLP model generates alternative for the missing values after fully training;

Step 3: $D_{com}$ and $D_{miss}$ are combined to recover the origin dataset, and $D_{miss}$ would also be a complete dataset after Step 2;

Step 4: The imputation performance is measured by using different imputation methods, such as the mode, random, KNN, and AE.

### 3.2. The Imputation Scheme Based on IMLP

Briefly, the imputation scheme based on IMLP (ISB-IMLP) includes four steps, i.e., determination of the missing types, construction of the MLP, training of the model, and reconstruction of the incomplete dataset. The details of the IMLP model scheme are expounded on below.

#### 3.2.1. The Determination of the Missing Types

**Figure 4:**
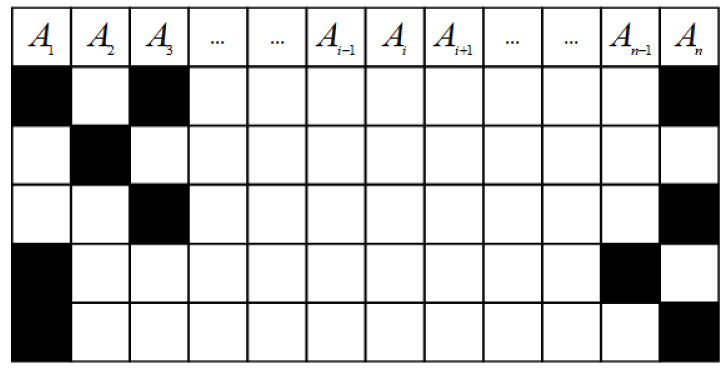
An example of five missing types.

The core methodology is to make use of the complete data to train the IMLP model and then predict the missing values utilizing the trained model. To conduct the simulation experiments, levels of noise are first artificially added to the object datasets; five different missing proportions are considered in this paper. Additionally, because there are three missing patterns (MAR, MCAR, and NMAR), each missing pattern is also artificially simulated according to its definition in this study. As shown in the research framework, the subset $D_{com}$ is chosen to train the IMLP model, and it is discretized via the one-hot encoding technique. According to the definition of missing mechanisms, there are usually multiple missing types in $D_{miss}$. Consequently, it is essential to determine the missing types before employing the overall IMLP scheme. Figure 4 depicts an example with five missing types, where $A_{i}\left(i=1,2,\ldots ,n\right)$ indicates the ith attribute of the incomplete dataset with n attributes. Moreover, the grid squares marked in black indicate the positions where the missing values appear.

Specifically, each row denotes an instance in the incomplete dataset. For the first instance, the black mark appears in $A_{1}$, $A_{2}$, and $A_{3}$, which means this kind of missing type can be denoted as $mt_{1}:\left\{1,2,n\right\}$; in the second instance, the missing value is shown at $A_{2}$; the third instance includes two missing values whose positions are $A_{3}$ and $A_{n}$. Figure 4 is just a basic example to illustrate the principle of identifying the missing type. The missing types of the object dataset for experiments can be determined based on the above method. As a result, the collection $MT=\left\{mt_{1},mt_{2},\ldots ,mt_{n}\right\}$ denotes all n missing types in $D_{miss}$. Note that all the illustrations are based on this example in this section.

#### 3.2.2. The Construction of the IMLP

The input space of the multilayer perceptron corresponds to the feature space of the model input data, and the number of neurons in the input layer is equal to the feature dimensions of the input data. The neurons in the input layers are fully connected to other neurons in the next layer. The last layer in the MLP network is the output layer, whose dimensions correspond to the feature dimensions of the model output data. The neurons in the output layer are also fully connected to the neurons in the previous layer. The network layers between the input and output layers are called hidden layers. On the one hand, the MLP method commonly makes use of the backpropagation algorithm to figure out all the parameters. However, the full connection architecture leads to considerable parameters and gradient descent computations, which require consumption of time and resources. On the other hand, according to the principles of the neural network, all the connection weights and biases in the network structure are calculated and updated by a gradient descent algorithm, which may be limited by a local optimum. All the above concerns are our motivations to propose the IMLP method.

In this study, we firstly construct an MLP model to fill in discrete missing values. The activation function in the hidden layers is $relu\left(x\right)$, and $sigmoid\left(x\right)$ is for the output layer. The input and output data are explained in the next subsection. The MLP model cannot straightforwardly deal with missing values, so prefilling the missing values through some specific approaches can not only allow more data to enter the model for the training on missing types, but also speed up the convergence. Strategically, the gradient descent with momentum is introduced to improve the performance of the MLP.

#### 3.2.3. The Training of the Model

Because there is a set of string data in the object datasets, they may not be trained well by the standard MLP model without encoding manipulation. In addition, some attribute values are denoted as integers, but they are encoded from discrete data whose actual meanings are discrete. Generally, if a specific model directly trains the continuous data without processing to fill in the missing values, float results will be obtained as the filling values. However, the filling values obtained via the above method cannot find the corresponding discrete patterns, which means the results do not satisfy the actual demand. Therefore, before the model training starts, the input data are encoded by the one-hot technique.

Additionally, some prefilling strategies are needed to maintain the data size for model training, as the data size for deep learning is crucial to the training performance. To the best of our knowledge, imputing the missing values via statistical techniques without calculating distances or similarities between different samples has an agreeable performance for both accuracy and time cost. Compared with MAR and NMAR, the missing data arise without relevance to the observed or unobserved features in MCAR. In this study, the mode method is used as the prefilling strategy for the missing pattern of both MAR and NMAR, and the random method is selected to prefill the missing values in MCAR.

**Figure 5:**
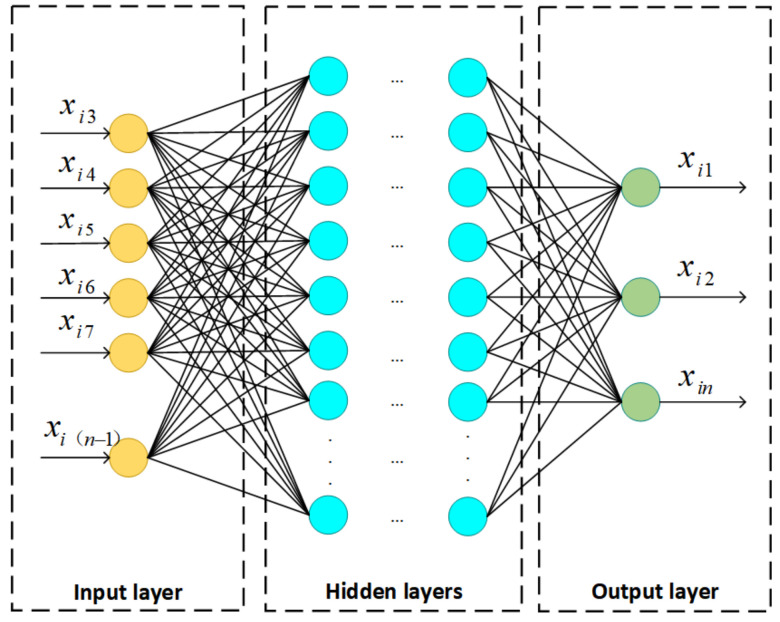
IMLP model for $mt_{i}$.

Figure 5 illustrates the overall architecture of the IMLP model. $mt_{1}$ is selected as an example of a missing type in Figure 4. Its missing positions are 1, 2, and n, which corresponds to the situation that data has missing values in the first, second, and last attributes. The IMLP model uses a complete dataset for training, in which features without missing values are the inputs of the model, and attributes corresponding to missing types are the outputs of the model. As shown in Figure 5, $x_{i}$ represents the ith sample in the object dataset. The elements in $\left\{x_{i3},x_{i4},\ldots ,x_{i\left(n-1\right)}\right\}$ are the input, while $x_{1}$, $x_{2}$, and $x_{n}$ are the output of the model. During the training process, the binary cross-entropy and momentum gradient descent algorithms are selected as the loss function and the optimizing strategy, respectively, to figure out the optimal parameters of the IMLP model. In a word, the fundamental law of the training is to utilize the observed attributes as input and the unobserved attributes as output to find the optimal network weights and biases, and each missing type can be trained similarly.

#### 3.2.4. The Reconstruction of the Incomplete Dataset

During this phase, the main target is to utilize the trained IMLP model to predict the missing attribute values and fill them with the predicted results. Specifically, the first step is to determine all the missing types of the target dataset. The model then provides the corresponding IMLP model for the defined missing type, which makes use of the observed data to impute the unobserved data. The incomplete data will then be filled in one by one. Finally, the model outputs a complete dataset for subsequent operations.

**Figure 6:**
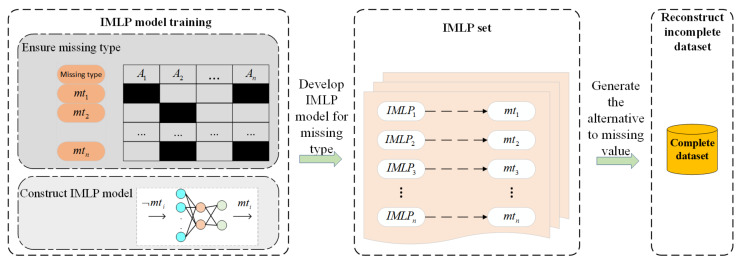
Overall process of ISB-IMLP.

In brief, the entire scheme can be illustrated in Figure 6, and the steps for filling in discrete missing data can be concisely summarized as follows.

First, the preliminary task is to determine the specific missing types for model development and training. The one-hot technique encodes the object data for discretizing. Each IMLP model is developed according to a specific missing type after data preprocessing. As a result, there is an IMLP set for imputing discrete missing data, where each model corresponds to a specific missing type. In fact, the discrete missing data imputation is regarded as a classification task in this paper. As for reconstructing the incomplete dataset, the alternatives to missing data are generated according to the IMLP set. Finally, the object dataset with missing data will be transformed into a complete dataset based on this scheme.

## 4. Experiments and Discussions

In this section, seven datasets collected from the UCI Machine Learning Repository are selected to verify the performance of different imputation methods with regard to classification accuracy. Additionally, the impacts of missing rates and missing mechanisms on imputation methods are also studied. Specifically, each dataset is conducted with three missing patterns (MAR, MCAR, and NMAR) and seven missing rates ranging from 10% to 30%, whose interval is 5%. In particular, the imputation performance of ISB-IMLP is compared with some standard imputation methods, i.e., the mode, random, and hot-deck imputation methods, as well as the imputation methods based on the k-nearest neighbors (KNN), decision tree (DT), random forest (RF), standard multilayer perceptron (MLP), and autoencoder (AE) techniques. Furthermore, the performances of the benchmark methods and the IMLP method on different missing patterns are evaluated, and the changing trends with different missing ratios are also researched. The performance evaluation is particularly performed based on classification accuracy by using several different classifiers on each dataset.

### 4.1. Experiment Setup

In this section, the details of the simulation experiments are represented in three aspects, i.e., platform, datasets, and other settings.

#### 4.1.1. Experiment Platform

The experiments in the paper are based on a simulation platform, whose experimental settings are as follows: Windows 10, 64-bit operating system, AMD R7-4800H process, and 16 GB RAM. The programming language is Python 3.7, and the main libraries used are NumPy 1.21, Pandas 1.3.4, scikit-learn 1.0.2, Keras 2.8, and TensorFlow 2.8.

#### 4.1.2. Dataset Description

Table 1 shows the characteristics of the datasets used in this section, including the number of data samples, attributes, and classes. These datasets are collected from different fields in the real world for binary or multi-class classification tasks, and they are all composed of discrete data or mixtures of discrete and continuous data. Particularly, there are some datasets in this table with missing values (i.e., Breast Cancer and Blood), which may reduce the effectiveness of the imputation method. Therefore, the pre-task is to remove the incomplete samples and obtain seven complete datasets. To simulate incomplete datasets and validate the performance of the proposed imputation method, each complete dataset is transformed into 15 variant datasets (three kinds of mechanisms, five kinds of missing rates). In this setting, it is convenient to evaluate the performance of imputation methods. Specifically, the complete dataset before deletion processing can be used as the control group, and the dataset after filling in the missing values can be used as the experimental group.

#### 4.1.3. Other Settings

As for settings for levels of missing proportions, five levels of noise are artificially added to the datasets in Table 1, ranging from 10% to 30%. Actually, deleting the missing data with smaller missing proportions may be the most efficient manipulation. Higher missing proportions may be improper for our object datasets and insufficient for model training.

As for settings for experimental methods, the random method stochastically selects an observed value as the filling alternative, and the mode method statistically sets the most frequent value as the imputing option. Both of them are without extra settings. The hot-deck method aims to find a substitution for the missing value based on the similarity between different samples. For the parameters of the machine learning technique, the number of neighbors for KNN is set to 5, and the Hamming distance is chosen to measure the nearest neighbors. Those methods based on the decision tree and random forest model classifiers develop models between observed attributes and unobserved attributes, which regard missing value imputation as a classification task. Generally, their parameters are the defaults that scikit-learn provides. In particular, the decision tree technique experimented with is CART, which employs the Gini coefficient as the dividing evidence. For the parameters of the neural network, all the object neural networks are composed of three hidden layers, where each layer has 32 neurons, and the learning rate, batch size, and epochs are set as 0.001, 256, and 1000 respectively. We adapt stochastic gradient descent with momentum as the optimizer and set the momentum coefficient as 0.9. The prefilling method for IMLP is the mode imputation method in both MAR and NMAR, and the random in MCAR.

On the other hand, classifiers used to evaluate the performance may bring biases, which may be related to the characteristics of the datasets or the distribution of the data. All the other hyper-parameters are set to their default values. In [11], some learning algorithms, including © Bayes (NB), k-nearest neighbors (KNN), and support vector machine (SVM), were considered to have biases on some specific datasets or data. In addition, the decision tree (DT) method has good performance for multi-class tasks. Therefore, NB, KNN, SVM, and DT are selected as different classifiers to verify the robustness of the imputation methods.

### 4.2. Experiment Analysis

#### 4.2.1. The Performance of Missing Value Imputation

According to the research framework in Section 3, this section aims to execute the last research phase, i.e., measure and evaluate the performance of different imputation methods compared with the imputation scheme based on IMLP (ISB-IMLP). Some statistical or machine learning techniques, including the mode, random, hot-deck, KNN, decision tree, random forest, MLP, and AE methods, are selected to fill the missing data as a control group. To evaluate the imputation performance of the different methods, classification accuracy based on the SVM classifier for the seven real-world datasets under three missing mechanisms is the primary evaluation metric. Note that all the results are average accuracies after 10-fold cross-validation, where the training and testing set ratio is 9:1. To eliminate the chance of erroneous results, the standard deviations of the five-times classification task are calculated and placed after the accuracy. All the results are presented in decimal form to simplify the calculation and analysis. Moreover, the classification accuracy based on the origin dataset without adding any noise is also obtained in comparison with the experimental method. In this section, SVM is selected as the learning algorithm for classification.

**Figure 7:**
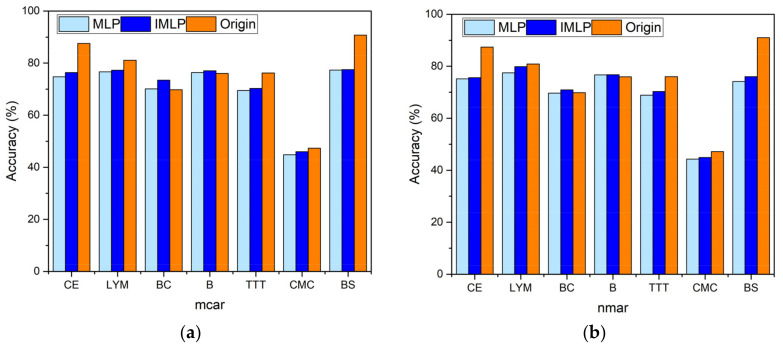
Average classification accuracy obtained from MLP, IMLP imputation methods and unprocessed dataset: (**a**) in MCAR and (**b**) in NMAR.

Table 2 presents the average classification accuracy with five missing proportions obtained based on the MLP and IMLP imputation models, where the experiments are conducted in MAR. The last row in this table represents the accuracy obtained from the clean dataset with the same operation as the others. Figure 7 visualizes the comparisons of accuracy obtained from MLP, IMLP, and the clean dataset in MCAR and NMAR. The accuracy obtained from the clean dataset acts as a standard for the others, where the closer to it, the better performance is. On the one hand, the classification accuracy obtained from the IMLP model is about 0.01 or 0.02 higher than that of the MLP model. This shows that our modification improved the model’s ability to fill in discrete missing data compared with the standard MLP model. On the other hand, the average classification accuracy is also close to the accuracy obtained from the unprocessed dataset.

When the object dataset is Breast Cancer or Blood, both IMLP and MLP have higher accuracy than the clean dataset, which contains missing data at first. This means that filling in missing data has a positive effect on improving performance. When the experimental dataset is Car Evaluation or Balance Scale, the difference from the standard accuracy is higher than for the other sets, which means that missing data significantly downgrade the classification performance in such a dataset. Generally, IMLP provides a better ability to fill in missing data comprehensively, which shows that the momentum descent algorithm and prefilling strategy have a positive effect on optimizing the performance of MLP.

Table 3 shows the complete experimental results for the scenario of MAR, while Table 4 and Table 5 present each imputation method’s average accuracy for five missing proportions on the object dataset for MCAR and NMAR, respectively. For Table 3, each row contains nine accuracies and standard deviations in a certain missing proportion for a specific dataset, where the bold represents the best accuracy obtained by the corresponding imputation method for each dataset with specific missing proportion. For Table 4 and Table 5, each row contains the accuracies obtained from different datasets based on the corresponding imputation method, where the bold represents the best classification accuracy for each dataset. 

MAR means that the missing values occur randomly, which is related to the observed complete samples or attribute values. In this regard, statistical or machine learning techniques are feasible for imputing missing values in theory. From this situation, Table 2 indicates that ISB-IMLP has good performance in most circumstances, especially for the Lymphography, Breast Cancer, Blood, and Contraceptive Method Choice datasets. As for the other test objects, the classification accuracy acquired from the proposed method is closer to the best one than the others. For example, when the object dataset is Car Evaluation, the mode imputation approach outperforms the others at the missing proportions of 0.1, 0.15, and 0.3. However, our method is as close as possible, and the corresponding differences are only 0.0057, 0.0044, and 0.0052. For the 35 sets of experimental results, the proposed method is 54.29% accurate in these cases against the 11.43% accuracy of its best competitor mode and KNN.

Gathering the results obtained using five missing proportions and seven datasets, the best, second-best, and worst average accuracy values are 71.52%, 70.04%, and 68.95%, respectively, and the corresponding methods are IMLP, decision tree, and KNN. Although imputing missing data via KNN obtained great performance for continuous data in much of the previous literature [12,13,30], the method’s ability to handle discrete missing data is not as good as its ability to handle continuous data. According to the results, IMLP brings 1.48% improvement compared with CART, and it was found that CART was suitable for discrete missing data in [26]. Because this statistical technique has low computational and training costs, filling in discrete missing data via this statistical technique is faster than schemes based on machine learning or deep learning techniques. Compared to the other experimental techniques, our imputation approach has computational cost for good training. However, IMLP provides overall superior classification performance. The combination of the statistical imputation technique and gradient descent algorithm makes models converge, providing better classification performance after filling in missing discrete data. The IMLP model performs better compared with the imputation scheme based on the standard MLP model, with the average classification accuracy of the IMLP model being 0.0177 higher. This indicates that IMLP provides improvements to MLP. Generally, it verifies that the IMLP model with a prefilling operation has better imputation performance in MAR.

MCAR means the missing values occur completely randomly, while NMAR refers to the case in which missing values are related to unobserved attribute values. For the case of the missing patterns MCAR and NMAR, Table 4 and Table 5 show an average performance on seven datasets of five levels of noise, respectively. As shown in Table 4, our method obtains the best classification results except in the case of the Lymphography dataset, which testifies that the proposed approach is also good for the MCAR missing pattern. Moreover, imputing missing values with the KNN technique also has good performance in this simulation situation. Especially for the Lymphography dataset, the KNN imputation method obtains the best classification accuracy. To statistically evaluate the improvement of ISB-IMLP, the average aggregating accuracy on seven datasets obtained from the MLP and IMLP models are 0.6991 and 0.7111, respectively; the latter average accuracy is 1.2% higher than the former.

As shown in Table 5, ISB-IMLP outperforms the other eight imputation methods by 71.43%. Though there are two excluded datasets, i.e., Blood and Balance Scale, their accuracies handled with the proposed method rank second only to the best one. Concretely, their differences from the best one are only 0.0004 and 0.0027, respectively. However, the best imputation methods for the Blood and Balance Scale datasets are mode and decision tree. On the one hand, these two methods are also acceptable for the experimental datasets in this missing pattern. On the other hand, our approach still has a better overall performance in NMAR.

**Figure 8:**
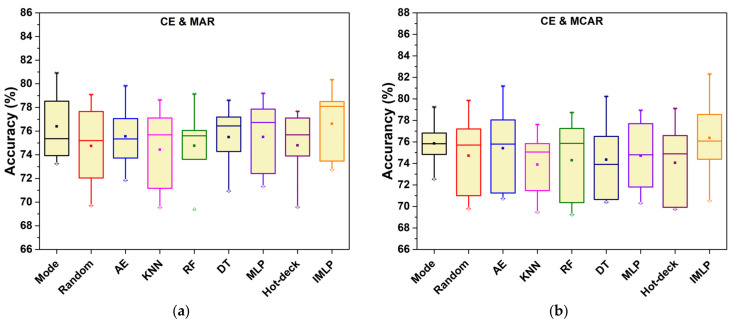

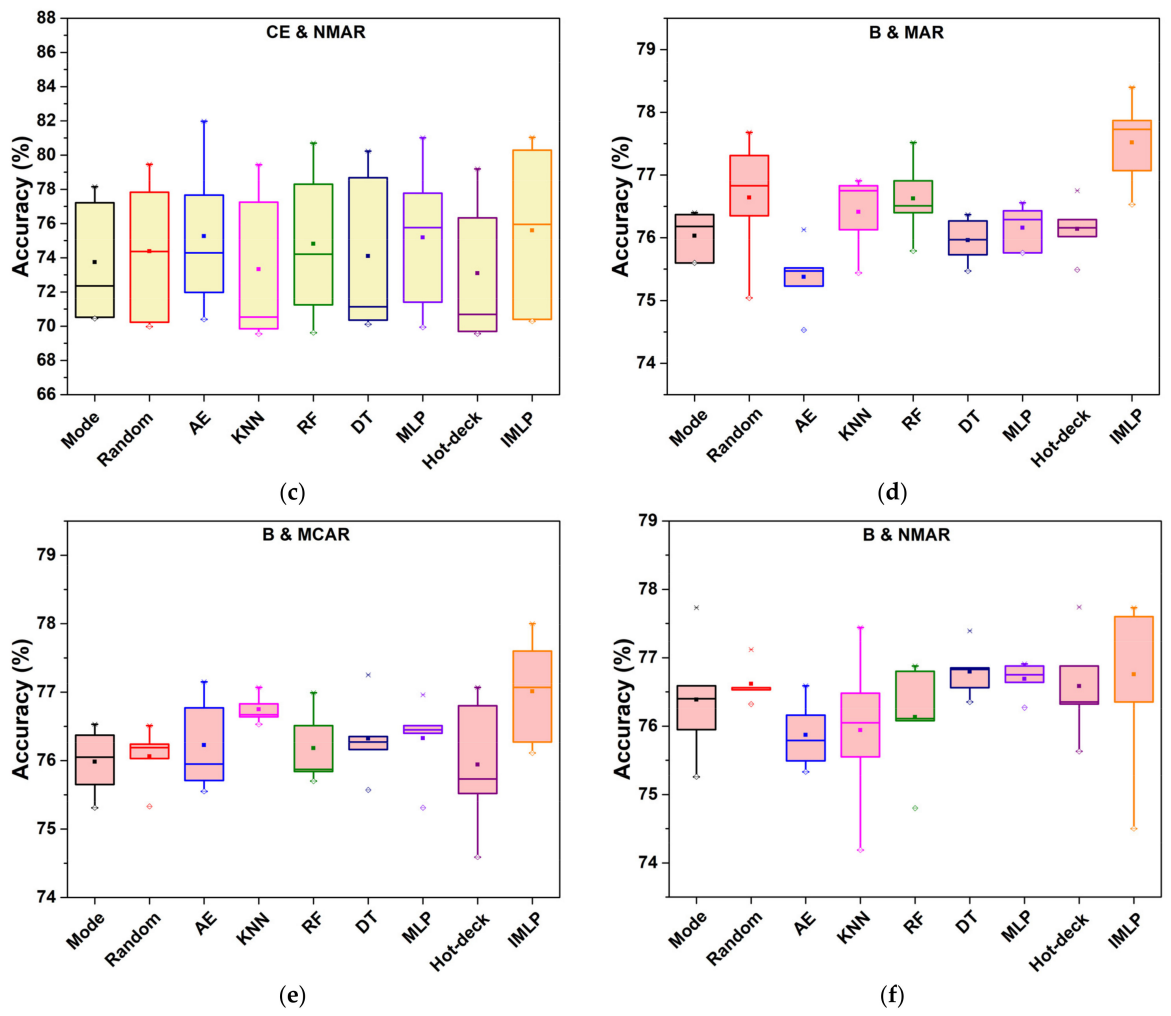
Boxplot of accuracies for five missing proportions for the Car Evaluation and Blood datasets in MAR, MCAR, and NMAR. (**a**–**c**) for the Car Evaluation dataset in MAR, MCAR, and NMAR, respectively. (**d**–**f**) for the Blood dataset in MAR, MCAR, and NMAR, respectively. For the element of boxplot, the solid square is the position of mean value, the hollow diamond is the position of 1%, and × represents the 99% of the box.

To illustrate stability visually, the boxplots indicate the classification accuracies obtained from nine experimental methods in Figure 8. The object datasets are Car Evaluation and Blood. According to the boxplots, ISB-IMLP has a longer box than the others in most situations. However, its mean, maximum, and minimum are comprehensively higher than those of the other methods. This indicates that the missing proportion affects the imputation performance. Moreover, the results show the specific standard deviation for the corresponding situation after the symbol ‘±’. In general, the proposed method may have no lowest standard deviation in most cases. Comparing the imputation methods based on statistical and machine learning techniques, the standard deviations of the methods based on deep learning methods (i.e., AE, MLP, and IMLP) are always higher than the two others, for which the reason may be the sample size or the distribution of data. Actually, these machine learning techniques with neural networks are sensitive to the scale of the training samples; insufficient training data will lead to underfitting. Compared with the standard MLP model, the results of our method are smooth as a whole.

**Figure 9:**
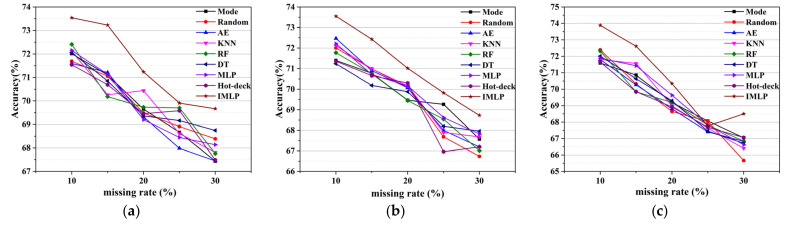
Average classification accuracies of SVM for different imputation models in three missing patterns: (**a**) in MAR; (**b**) in MCAR; (**c**) in NMAR.

In [11,33], the average classification accuracy obtained utilizing different imputation methods with different missing rates was selected to show an overall changing trend of continuous missing data. Figure 9 exhibits the average SVM classification accuracy based on different imputation models, where the *x*-axis and *y*-axis represent missing rates and average accuracy for each line chart, respectively. A higher missing rate means more missing information for the classification learning algorithm. Thus, the average accuracy decreases as the missing proportion increases, regardless of the imputation method. However, there are several excluded cases in which the average accuracy increases with the addition of the missing proportion. For example, when the missing ratio is 30% in NMAR, the accuracy of the proposed method increases compared to the accuracy of the 25% missing proportion. One reason could be that the artificial operation for filling missing values impacts the classification task positively. In particular, the accuracy in MAR and MCAR is around 2% higher than the accuracy in NMAR, which means that it may be harder to deal with the missing data in NMAR. As a whole, our method outperforms many classical imputation methods for discrete missing data, according to these line charts. Though the mode and random methods have fast speeds of convergence for discrete missing data imputation, ISB-IMLP gives a robust and higher classification accuracy for datasets with discrete missing data, albeit with a larger time cost. As for schemes for filling discrete missing data based on model learning, ISB-IMLP optimizes the convergence of the model and provides stable performance for each missing mechanism.

#### 4.2.2. The Imputation Performance of Different Classifiers

**Figure 10:**
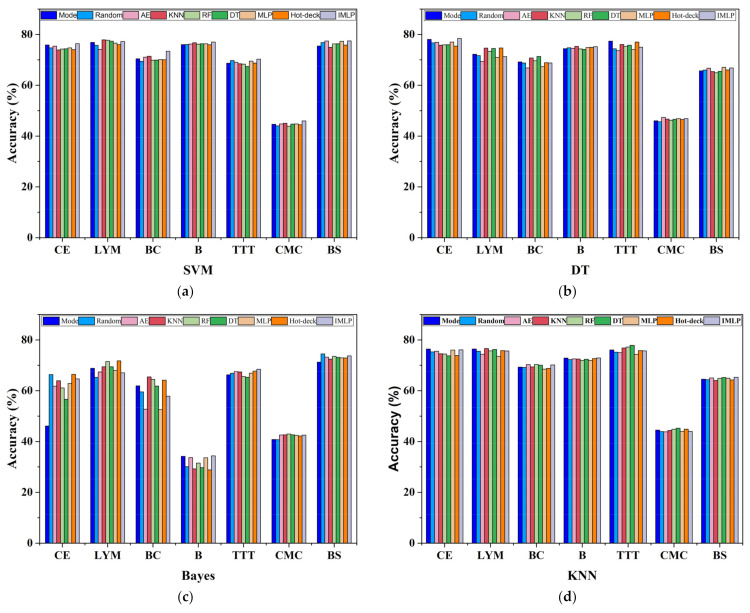
Performance analysis of four classifiers for nine imputation methods in MCAR. (**a**) for SVM; (**b**) for decision tree; (**c**) for naïve Bayes; and (**d**) for KNN.

The selection of the classification learning algorithm always affects the final performance. Consequently, many studies aim to investigate the influence of the choice of different classifiers on imputation performance [11,40,51]. Figure 10 presents the integrated average accuracy for five missing proportions in the missing mechanism of MCAR. As for the reason for evaluating in the case of such a missing mechanism, the missing value appears completely at random, which may be unstable in imputation performance. The *x*-axis is the name of the object datasets, while the *y*-axis is the aggregated accuracy. Our proposed method does not necessarily achieve the best accuracy overall according to the histogram, which means it is advisable to select a concrete learning algorithm for specific datasets. Specifically, the Naive Bayes classifier may not be the first choice for the Car Evaluation dataset, where its overall accuracy is lower than 70%, while the other three obtain accuracies over 70%. Moreover, all the imputation methods using the Naive Bayes learning algorithm for the Blood dataset have low accuracies, and the others are 30% higher. It is also evident that the SVM and Naive Bayes models are more suitable for the Balance Scale dataset, where their accuracies are about 5% higher than the accuracies acquired by the decision tree and KNN models. Note that ISB-IMLP with a decision tree for classification has lower superiority than the others in datasets with small sizes of feature numbers, where the distribution of the dataset has a negative effect on ISB-IMLP. In this regard, the selection of classifiers plays a significant role in classification tasks. On the other hand, it is found that our method combined with SVM can obtain the overall best performance; an object dataset with a small number of samples, where the missing values are filled in by the imputation methods using statistical techniques, is generally better than the proposed method. One reason could be the deficiency of the training sample, which might not converge in the finite iterations. Consequently, it is also necessary to determine suitable methods for datasets of different sizes.

However, it is difficult to conclude which learning algorithm and imputation method perform the best. The reasons can be summarized as follows. First, the uncertainty of data distribution and the difficulty of determining it affect the imputation performance for discrete data. Second, different learning algorithms have distinctive biases on the final results, which would lead to a high difference between the two classifiers. Moreover, the missing pattern might be another factor affecting the results. In conclusion, our modified MLP model can reach acceptable performance in most situations for discrete missing values, which could enhance imputation theory in the future.

## 5. Conclusions

The paper concentrates on developing an approach to fill in discrete missing data and applying it to real-world classification tasks; an imputation scheme based on IMLP (ISB-IMLP) is proposed. Specifically, the standard MLP method combined with gradient descent and definite prefilling plans is regarded as an approach for filling in discrete missing data. ISB-IMLP first develops classification models for each missing type, and the generated alternatives to missing types are gathered to reconstruct the incomplete dataset. To explore the effect of missing mechanisms, missing proportions, and selection of the learning algorithm, the performance of ISB-IMLP is evaluated through experiments, which are conducted on seven real-world datasets under three missing mechanisms (MAR, MCAR, and NMAR); the results are compared with those of eight typical imputation methods. Moreover, experimental comparisons were made using five missing proportions ranging from 10% to 30%. The baseline imputation methods were the mode, random, hot-deck, KNN, decision tree, random forest, autoencoder, and standard MLP techniques. The simulation shows that ISB-IMLP has superior performance to MLP for each missing mechanism, with the former’s classification accuracy being around 1% or 2% higher than the latter’s. Compared to the statistics-based methods, ISB-IMLP has positive effects on imputation performance without consideration of the time cost. According to the results, it is a challenge to find a general method for missing situations. The reasons for this issue may be considered as the missing mechanism and the missing proportion, the selection of the classification algorithm, and the data size for model development. As a whole, ISB-IMLP performs well under three missing mechanisms in most situations, offering a practical approach for discrete missing data.

There are several unresolved issues that could be researched further in the future. First, it is difficult to find an imputation method applicable to all missing mechanisms, whether based on statistical theory or machine learning techniques, which will be one of the main tasks of future missing value imputation research. Moreover, missing values affect the classification accuracy and the efficiency of data processing techniques such as feature selection. Given the above analysis, missing value imputation has several challenges to overcome in the future and has extensive research implications.

## Figures and Tables

**Table 1:** Details of UCI datasets used in the experiments.

Dataset Name	No. of Samples	No. of Features	No. of Classes
Blood (B)	748	5	2
Breast Cancer (BC)	286	9	2
Balance Scale (BS)	625	4	5
Car Evaluation (CE)	1728	6	4
Contraceptive Method choice (CMC)	1473	9	3
Lymphography (LYM)	148	18	4
Tic-Tac-Toe (TTT)	958	9	2

**Table 2:** Average classification accuracy obtained from MLP, IMLP imputation methods and unprocessed dataset in MAR.

	CE	LYM	BC	B	TTT	CMC	BS
MLP	0.75506	0.74668	0.71074	0.7616	0.700675	0.42888	0.77778
IMLP	0.76626	0.78508	0.7262	0.7752	0.72215	0.44054	0.7887
Origin	0.875816	0.806664	0.70234	0.761924	0.76434	0.471	0.910028

**Table 3:** Classification accuracy and standard deviation with SVM classifier on seven datasets in MAR missing pattern.

Dataset	Rates	Method								
		Mode	Random	AE	KNN	RF	DT	MLP	Hot-Deck	IMLP
CE	0.1	**0.8092 ± 0.0064**	0.7909 ± 0.0088	0.7983 ± 0.0064	0.7864 ± 0.0109	0.7914 ± 0.014	0.7861 ± 0.0046	0.792 ± 0.0133	0.7768 ± 0.0137	0.8035 ± 0.0127
	0.15	**0.7853 ± 0.0084**	0.7766 ± 0.0093	0.7706 ± 0.0127	0.7711 ± 0.0146	0.7605 ± 0.0056	0.7719 ± 0.0093	0.7786 ± 0.0136	0.7711 ± 0.0101	0.7809 ± 0.0104
	0.2	0.7536 ± 0.0055	0.752 ± 0.0063	0.7534 ± 0.0133	0.7569 ± 0.0113	0.7561 ± 0.0096	0.7644 ± 0.0118	0.7673 ± 0.015	0.7569 ± 0.003	**0.785 ± 0.0217**
	0.25	0.7393 ± 0.0159	0.7204 ± 0.0206	0.7373 ± 0.013	0.6953 ± 0.006	0.7362 ± 0.0037	**0.7428 ± 0.0075**	0.7242 ± 0.0206	0.739 ± 0.0145	0.7347 ± 0.006
	0.3	**0.7324 ± 0.0075**	0.6971 ± 0.013	0.7184 ± 0.0155	0.7117 ± 0.0152	0.6938 ± 0.0096	0.7093 ± 0.013	0.7132 ± 0.0135	0.6957 ± 0.012	0.7272 ± 0.0126
LYM	0.1	0.7453 ± 0.0644	0.776 ± 0.0153	0.7746 ± 0.0296	0.792 ± 0.078	**0.8227 ± 0.0167**	0.8053 ± 0.0338	0.7747 ± 0.0506	0.7733 ± 0.0323	0.78 ± 0.0145
	0.15	0.7387 ± 0.0233	0.7814 ± 0.0578	0.8027 ± 0.0204	0.7587 ± 0.026	0.784 ± 0.0238	0.7987 ± 0.0247	0.7773 ± 0.0243	0.8067 ± 0.0302	**0.8267 ± 0.0352**
	0.2	0.7547 ± 0.0357	0.7267 ± 0.0133	0.72 ± 0.046	**0.7947 ± 0.0328**	0.7933 ± 0.0236	0.7533 ± 0.0383	0.728 ± 0.0331	0.7667 ± 0.038	0.7587 ± 0.0159
	0.25	0.7213 ± 0.0443	0.74 ± 0.0531	0.6987 ± 0.0369	0.7587 ± 0.0242	0.7773 ± 0.0555	0.7653 ± 0.0563	0.724 ± 0.0243	0.7413 ± 0.042	**0.78 ± 0.0293**
	0.3	0.6667 ± 0.0403	0.7387 ± 0.0441	0.7093 ± 0.0454	0.7347 ± 0.0128	0.736 ± 0.0423	0.7773 ± 0.0586	0.7294 ± 0.0473	0.7347 ± 0.0272	**0.78 ± 0.0445**
BC	0.1	0.7179 ± 0.0396	0.7062 ± 0.0321	0.6848 ± 0.0202	0.7014 ± 0.0267	0.7069 ± 0.0261	0.7028 ± 0.0209	0.7124 ± 0.0273	0.7145 ± 0.0222	**0.7483 ± 0.0317**
	0.15	0.7241 ± 0.0176	0.7131 ± 0.0324	0.72 ± 0.0215	0.7028 ± 0.0207	0.6917 ± 0.0318	0.7083 ± 0.0209	0.6993 ± 0.0247	0.689 ± 0.0311	**0.7345 ± 0.0252**
	0.2	0.6966 ± 0.0177	0.7034 ± 0.0205	0.7055 ± 0.0254	0.6993 ± 0.0136	0.6924 ± 0.0247	0.7007 ± 0.0257	0.6993 ± 0.0367	0.6856 ± 0.0079	**0.7241 ± 0.0343**
	0.25	0.6938 ± 0.0268	0.7152 ± 0.0259	0.6917 ± 0.0072	0.7021 ± 0.0436	0.7124 ± 0.0149	0.7083 ± 0.027	0.7103 ± 0.0144	**0.7255 ± 0.0246**	0.7069 ± 0.0155
	0.3	0.7 ± 0.0311	0.7076 ± 0.0206	0.6986 ± 0.0219	0.7028 ± 0.0263	0.7055 ± 0.0262	0.7083 ± 0.0328	**0.7324 ± 0.0135**	0.6862 ± 0.0195	0.7172 ± 0.0214
B	0.1	0.764 ± 0.0107	0.7635 ± 0.0116	0.7552 ± 0.0128	0.7675 ± 0.007	0.7691 ± 0.0121	0.7627 ± 0.0116	0.7576 ± 0.0099	0.7616 ± 0.0143	**0.7773 ± 0.0116**
	0.15	0.7618 ± 0.0214	0.7504 ± 0.0136	0.7523 ± 0.0092	0.7613 ± 0.0069	0.7579 ± 0.0058	0.7573 ± 0.012	0.7629 ± 0.0149	0.7549 ± 0.0093	**0.784 ± 0.0142**
	0.2	0.756 ± 0.0178	**0.7768 ± 0.0201**	0.7453 ± 0.0174	0.7691 ± 0.0141	0.764 ± 0.0172	0.7597 ± 0.0134	0.7656 ± 0.0138	0.7602 ± 0.0177	0.7653 ± 0.0119
	0.25	0.7637 ± 0.0061	0.7683 ± 0.0224	0.7613 ± 0.0154	0.7544 ± 0.0105	0.7651 ± 0.0125	0.7547 ± 0.016	0.7643 ± 0.015	0.7675 ± 0.0133	**0.7707 ± 0.0137**
	0.3	0.756 ± 0.012	0.7731 ± 0.0108	0.7547 ± 0.0106	0.7683 ± 0.0149	0.7752 ± 0.012	0.7637 ± 0.0109	0.7576 ± 0.0167	0.7629 ± 0.0168	**0.7787 ± 0.0133**
TTT	0.1	0.7281 ± 0.0056	0.7125 ± 0.0192	0.7167 ± 0.0134	**0.7369 ± 0.0107**	0.7304 ± 0.0179	0.6971 ± 0.0134	0.7288 ± 0.0198	0.7169 ± 0.0146	0.73235 ± 0.0144
	0.15	0.716 ± 0.0099	0.7021 ± 0.0233	0.7071 ± 0.0079	0.7156 ± 0.0099	0.6894 ± 0.011	0.6908 ± 0.0089	0.7127 ± 0.0072	0.715 ± 0.0102	**0.7344 ± 0.0293**
	0.2	0.699 ± 0.0193	0.7138 ± 0.0156	0.7065 ± 0.013	0.7152 ± 0.0208	0.6827 ± 0.0111	0.6933 ± 0.0155	0.6946 ± 0.0069	0.6965 ± 0.0164	**0.7312 ± 0.0229**
	0.25	0.7075 ± 0.0178	0.7056 ± 0.0124	0.7 ± 0.0078	0.7108 ± 0.0141	0.7017 ± 0.0213	0.6933 ± 0.0191	0.6981 ± 0.0183	**0.719 ± 0.0172**	0.7115 ± 0.014
	0.3	0.7083 ± 0.0186	**0.7177 ± 0.02**	0.6933 ± 0.0146	0.6975 ± 0.0084	0.6892 ± 0.0192	0.7021 ± 0.0119	0.6973 ± 0.0181	0.7029 ± 0.017	0.7115 ± 0.0132
CMC	0.1	0.4384 ± 0.0194	0.4382 ± 0.0119	0.4464 ± 0.0126	0.4478 ± 0.0098	0.4496 ± 0.016	0.4488 ± 0.0231	0.4355 ± 0.0137	0.4389 ± 0.0191	**0.4507 ± 0.004**
	0.15	0.4328 ± 0.0073	0.4428 ± 0.0153	0.4331 ± 0.0027	0.4295 ± 0.0206	0.431 ± 0.0121	0.4343 ± 0.012	0.4305 ± 0.0077	0.4285 ± 0.0136	**0.4574 ± 0.0135**
	0.2	0.4311 ± 0.0071	0.4321 ± 0.012	0.4305 ± 0.0142	0.4324 ± 0.008	0.4301 ± 0.0097	0.4228 ± 0.0153	0.4212 ± 0.0178	0.4282 ± 0.0115	**0.4405 ± 0.0155**
	0.25	0.4205 ± 0.018	0.4216 ± 0.0233	0.4211 ± 0.0074	**0.435 ± 0.0155**	0.4268 ± 0.0121	0.4343 ± 0.0159	0.428 ± 0.0084	0.4316 ± 0.0085	0.425 ± 0.0056
	0.3	0.4304 ± 0.0201	0.4293 ± 0.0084	0.4242 ± 0.0104	**0.4327 ± 0.0235**	0.4292 ± 0.0178	0.4209 ± 0.0132	0.4292 ± 0.0051	0.4315 ± 0.0097	0.4291 ± 0.0063
BS	0.1	0.8381 ± 0.0213	0.8311 ± 0.0118	0.8359 ± 0.0217	0.8162 ± 0.0201	0.7984 ± 0.0189	0.8412 ± 0.0172	0.8495 ± 0.0192	0.8257 ± 0.0066	**0.8556 ± 0.0178**
	0.15	**0.8187 ± 0.0155**	0.8076 ± 0.0182	0.799 ± 0.0238	0.7794 ± 0.019	0.7981 ± 0.0214	0.7984 ± 0.0194	0.8181 ± 0.0153	0.7832 ± 0.0127	0.8079 ± 0.0095
	0.2	0.7848 ± 0.0215	0.7594 ± 0.0149	**0.7914 ± 0.0189**	0.7632 ± 0.0146	0.7632 ± 0.0072	0.7616 ± 0.0184	0.7683 ± 0.0214	0.7682 ± 0.0151	0.7816 ± 0.012
	0.25	0.7607 ± 0.0107	0.7527 ± 0.0144	0.7489 ± 0.0308	0.7467 ± 0.0088	0.76 ± 0.0183	0.7429 ± 0.0101	0.7425 ± 0.0153	0.7473 ± 0.0125	**0.7651 ± 0.0287**
	0.3	0.727 ± 0.0128	0.7238 ± 0.0176	0.7229 ± 0.0257	0.6994 ± 0.0171	0.7143 ± 0.0135	0.7308 ± 0.0095	0.7105 ± 0.0203	0.7105 ± 0.0239	**0.7333 ± 0.0092**

**Table 4:** Average classification accuracy and standard deviation with SVM classifier on seven datasets in MCAR missing pattern.

	CE	LYM	BC	B	TTT	CMC	BS
**Mode**	0.75852 ± 0.0105	0.76852 ± 0.03464	0.70428 ± 0.03254	0.75982 ± 0.01632	0.68676 ± 0.01174	0.44646 ± 0.01356	0.75436 ± 0.0142
**Random**	0.74708 ± 0.01364	0.75734 ± 0.027	0.69408 ± 0.0228	0.7606 ± 0.01658	0.69762 ± 0.01782	0.44012 ± 0.0096	0.76818 ± 0.0202
**AE**	0.754 ± 0.0095	0.74132 ± 0.03356	0.71076 ± 0.01978	0.76226 ± 0.01348	0.69058 ± 0.01888	0.44796 ± 0.0138	0.77404 ± 0.0208
**KNN**	0.7389 ± 0.01006	**0.77866 ± 0.033**	0.71448 ± 0.02618	0.76748 ± 0.01314	0.6844 ± 0.01346	0.4506 ± 0.01284	0.74948 ± 0.0135
**RF**	0.74284 ± 0.00766	0.7776 ± 0.04184	0.69862 ± 0.02204	0.76182 ± 0.01234	0.68316 ± 0.0135	0.43884 ± 0.01288	0.7626 ± 0.01586
**DT**	0.74338 ± 0.0091	0.7736 ± 0.02096	0.69932 ± 0.02926	0.7632 ± 0.0148	0.67418 ± 0.01712	0.44734 ± 0.01258	0.76342 ± 0.0147
**MLP**	0.7471 ± 0.01264	0.76612 ± 0.0392	0.70124 ± 0.02072	0.76326 ± 0.01022	0.69496 ± 0.01782	0.44776 ± 0.01246	0.77308 ± 0.0116
**Hot-deck**	0.7405 ± 0.0091	0.76 ± 0.03058	0.70068 ± 0.02982	0.75942 ± 0.01426	0.68732 ± 0.01182	0.44484 ± 0.01014	0.75808 ± 0.0166
**IMLP**	**0.76368 ± 0.01534**	0.77252 ± 0.03774	**0.73378 ± 0.03006**	**0.7701 ± 0.01094**	**0.70292 ± 0.01976**	**0.45986 ± 0.01364**	**0.77484 ± 0.0145**

**Table 5:** Average classification accuracy and standard deviation with SVM classifier on seven datasets in NMAR missing pattern.

	CE	LYM	BC	B	TTT	CMC	BS
**Mode**	0.73736 ± 0.00922	0.73998 ± 0.03076	0.7044 ± 0.02292	0.76386 ± 0.01538	0.70026 ± 0.01642	0.44612 ± 0.01038	**0.7626 ± 0.01542**
**Random**	0.74374 ± 0.01044	0.74614 ± 0.03696	0.70606 ± 0.02442	0.76618 ± 0.01238	0.68308 ± 0.01988	0.44062 ± 0.01456	0.74516 ± 0.0152
**AE**	0.75258 ± 0.01218	0.76586 ± 0.0374	0.69972 ± 0.02182	0.75872 ± 0.01156	0.68342 ± 0.017	0.43874 ± 0.01234	0.7328 ± 0.02184
**KNN**	0.73324 ± 0.00816	0.79254 ± 0.03296	0.70554 ± 0.0279	0.75942 ± 0.01326	0.67988 ± 0.01466	0.4434 ± 0.01134	0.7356 ± 0.01344
**RF**	0.74816 ± 0.00878	0.77278 ± 0.0389	0.7 ± 0.02498	0.76134 ± 0.01668	0.6856 ± 0.01566	0.43828 ± 0.01296	0.7381 ± 0.01398
**DT**	0.74102 ± 0.01464	0.7792 ± 0.03738	0.7091 ± 0.02434	**0.76796 ± 0.01314**	0.67914 ± 0.0176	0.43882 ± 0.01094	0.73034 ± 0.015
**MLP**	0.75176 ± 0.0114	0.77494 ± 0.03936	0.69656 ± 0.02284	0.7669 ± 0.01362	0.68902 ± 0.01428	0.44292 ± 0.01292	0.7413 ± 0.01892
**Hot-deck**	0.73094 ± 0.01246	0.7864 ± 0.02186	0.7007 ± 0.02144	0.76584 ± 0.01586	0.6798 ± 0.01152	0.4472 ± 0.01442	0.7215 ± 0.01444
**IMLP**	**0.75594 ± 0.00996**	**0.79868 ± 0.03614**	**0.70936 ± 0.02064**	0.76756 ± 0.01534	**0.70302 ± 0.02056**	**0.44898 ± 0.0127**	0.7599 ± 0.01774

## Data Availability

Not applicable.
